# Effect of ginseng on fatigue related to neuromyelitis optica spectrum disorder: A double-blinded randomized controlled clinical trial

**DOI:** 10.22088/cjim.15.1.17

**Published:** 2024

**Authors:** Sara Aslzadeh, Shaghayegh Shahmirzaei, Mohammad Ali Sahraian, Razieh Sadat Kazemi Mozdabadi, Hossein Rezaei Aliabadi, Mohammad Reza Gheini, Nasim Rezaeimanesh, Sharareh Eskandarieh, Fazeleh Majidi, Abdorreza Naser Moghadasi

**Affiliations:** 1 Department of Neurology, Sina Hospital, Tehran University of Medical Sciences, Tehran, Iran; 2 Multiple Sclerosis Research Center, Neuroscience Institute, Tehran University of Medical Sciences, Tehran, Iran; 3 Bam University of Medical Sciences, Bam, Iran; 4 Research Development Center, Sina Hospital, Tehran University of Medical Sciences, Tehran, Iran

**Keywords:** Neuromyelitis optica spectrum disorder, Fatigue, Ginseng.

## Abstract

**Background:**

The effects of ginseng on fatigue have been proven in patients with multiple sclerosis (MS), which have several similar manifestations to neuromyelitis optica spectrum disorder (NMOSD) patients. This study was designed to evaluate the effects of ginseng on fatigue in NMOSD patients.

**Methods:**

In this double-blinded randomized controlled clinical trial, 64 patients were recruited and were allocated into two study groups (ginseng or placebo) via block randomization. The participants received either 250-mg ginseng or placebo twice daily for a 3-month period. Also, the measurement of outcome was performed using the valid and reliable Persian version of fatigue severity scale (FSS) questionnaire, which was filled by patients once after enrollment in the study and once at the end of the study post-intervention.

**Results:**

In total, 58 patients finished the study with no major side effects. There were no significant differences in demographic, clinical, as well as FSS between two study groups (p>0.05). Ginseng supplementation significantly reduced fatigue (40.21±13.51 vs. 28.97±14.18; p˂0.01), while patients in the placebo group showed significantly higher fatigue score after 3 months post-intervention (35.03±13.51 vs. 38.79±12.27; P: 0.02). The extent of changes in the fatigue score in the ginseng group was significantly greater than in the placebo group (p ˂0.01).

**Conclusion:**

This study revealed positive effects of ginseng on reducing fatigue in NMOSD patients with no major side effects. In this regard, further studies are warranted to evaluate and clarify the effects of ginseng on fatigue in NMOSD.

Neuromyelitis optica spectrum disorder (NMOSD), previously known as Devic disease, is an inflammatory disorder of the central nervous system, which mainly targets the optic nerves and spinal cord (1-3). Moreover, NMOSD can be distinguished from multiple sclerosis (MS) by discovering anti aquaporin-4 antibody (AQP4) (4). The prevalence of NMOSD was reported 0.86 per 100,000 person in Tehran, the capital city of Iran (5). A number of motor and psychological problems such as fatigue, hopelessness, and suicidality have been reported in NMOSD patients (6, 7). Correspondingly, fatigue is a major burden in patients with NMOSD, which can have a negative impact on the quality of life in patients. Between 50 and 71.4% of NMOSD patients experience fatigue, whose severity is correlated with decreased quality of sleep as well as increased severity of pain. Notably, it has been proven that the length of disease has a negative impact on fatigue (8, 9). 

To the best of our knowledge, there is only one study related to intervention for fatigue reduction in NMOSD, so far. Araki et al.’s study indicated that monthly injection of tocilizumab (8 mg/kg) reduces fatigue. Tocilizumab is a monoclonal antibody against the interleukin-6 receptor, which is prescribed for the treatment of NMOSD (10). So, it is very important to introduce other drugs that reduce fatigue in NMOSD.

Ginseng is a herbal medicine known for its effects on nervous, cardiovascular, reproductive, and metabolic systems (11). In addition, there are several studies showing improvement in fatigue status with ginseng (12). Further, the effects of ginseng on fatigue have been proven in patients with multiple sclerosis (MS), which have several similar manifestations to NMOSD patients (13-15). To the best of our knowledge, these effects have not been studied on NMOSD patients.

Studies suggest that excessive production of reactive oxygen species may cause chronic fatigue (16, 17). Thus, natural products such as ginseng may reduce fatigue due to their antioxidant characteristics (18). Accordingly, this study was designed and performed to assess the effect of ginseng on fatigue in NMOSD patients.

## Methods

**Study design and setting:** This investigation was a double-blinded randomized placebo-controlled parallel trial conducted on NMOSD patients in Sina hospital, Tehran, Iran, between 2018 and 2019. The protocol was designed by experts in clinical research related to NMOSD, aiming to assess the safety and efficacy of ginseng on the fatigue observed in NMOSD patients. 

**Ethical approval and informed consent:** The trial was registered in Iranian Registry of Clinical Trials with IRCT number IRCT20200713048095N1 and was then approved by the Ethics Committee of Tehran University of Medical Sciences (IR.TUMS.MEDICINE.REC.1398.969).

The study protocol was described for all the participants at the beginning of study and written informed consent form was completed. 

**Sample Size:** The sample size was calculated by “n = [(Z_α/2 _+ Z_β_)^2^ × {2(ó)^2^}]/ (μ1 - μ2)^2^” formula, using Etamadifar et al.’s study [μ2= -1.46, μ1= 8.04, SD: 12.9, 80% power, α:0.05; ] (13). By considering 20% loss rate, the sample size determined 32 patients in each group.

**Participants:** Patients who were referred to Sina Hospital NMOSD clinic of Tehran University of Medical Sciences, Tehran, Iran were enrolled using convenience sampling method if meeting the inclusion criteria. They were recruited in the trial by a senior neurology resident in Sina NMOSD clinic after obtaining written informed consent; they were randomly assigned 1:1 into either treatment or placebo group using block randomization (16 blocks with 4 patients in each block). The participants and researchers (who assessed the outcomes) were blind. Random allocation sequence was generated by a physician out of study. 

The inclusion criteria used in this study consisted of the 2015 diagnostic criteria for NMOSD (19). The exclusion criteria were as follows: 1. Pregnancy or breast feeding 2. Concomitant use of any anti-fatigue medications such as modafinil, amantadine and L-carnitine, etc. 3. Occurrence of life-threatening side effects 4. Having any concomitant diseases affecting fatigue.

**Data collection:** Demographic and clinical data including age, sex, past medical history, family history of NMOSD or MS, past medication history, and number of attacks during past year were collected. Expanded Disability Status Scale (EDSS) was calculated by an expert neurologist. Further, the severity of fatigue was assessed at the beginning of study and after 3 months of receiving supplementation using the Persian version of Fatigue Severity Scale (FSS). This scale is a valid and reliable self-report questionnaire containing nine items evaluating the severity of the impact of fatigue on daily life (20, 21). 

Correspondingly, each item is scored from 1 to 7 by the patient. A total score of 36 or higher would be considered as severe fatigue in these patients (20, 21). Administration of the questionnaires and calculations of scores were performed twice by a single neurologist. The included patients were followed through text messages every week for evaluating their medication adherence. The threshold of adherence was 80%, based on Haynes’ definition of adherence to antihypertensive medication as taking ≥80% of pills. 

If a patient would not respond to text messages, a phone call would have been made. All data collection and physical examinations were done by a single neurologist to minimize the inter-observer bias.

**Procedure:** The patients in the treatment group received ginseng 250-mg oral tablets twice daily for a 3-month period. On the other hand, the patients in the control group received resembling oral placebo tablets with the same instruction. The patients were provided with 30x2x3 tablets at once and were also educated and informed on the use as well as side effects by the same physician. The study physician was a senior neurology resident who was blinded to the groups and randomization coding. The patients were also blinded to the medication they took. Only the company providing the medication knew the codes. 

**Statistical analysis:** After reviewing the normality test, parametric methods were used to investigate the relationship between the variables. The independent sample t-test was used to compare the baseline quantitative characteristics and changes of FSS among ginseng and placebo groups. We also used Paired samples t-test to examine the difference of fatigue score before and after the intervention in each study group. The ANCOVA univariate analysis was employed to compare FSS post-intervention by adjusting for the base FSS score. The significance level was considered less than 0.05. All the above-mentioned analyses were performed using SPSS software Version 23.

## Results

**Figure 1:**
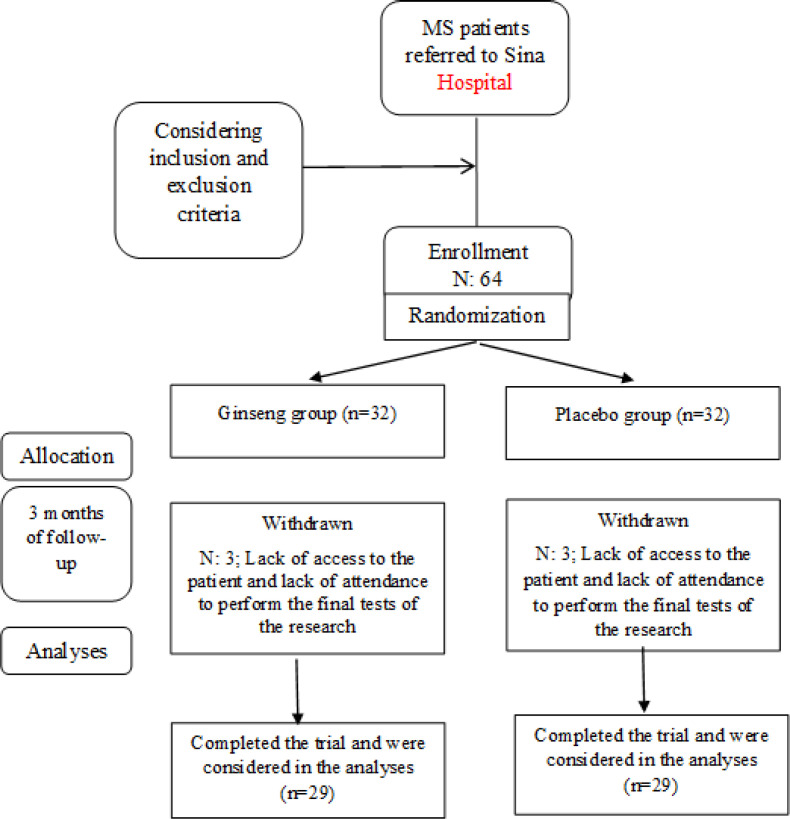
Study participants’ diagram

**Patient characteristics:** A total number of 58 patients who were previously definitely diagnosed with NMOSD completed the study and were enrolled in the analysis (figure 1).

 The baseline characteristics of the participants are summarized in table 1. The mean age of participants was 36.84 ± 9.37 years old, with 52 female subjects (89.7%). Notably, there was no statistical difference between the two groups for clinical and demographic characteristics (p>0.05). 

**Table 1:** Baseline demographic and clinical characteristics of patients

	**Intervention group**	**Placebo group**	**P-value**
**Age (years)**	35.4±8.7	38.2±9.9	0.24
**Duration of disease (years)**	7.4±4.5	6.2±4.4	0.34
**EDSS**	2.6±1.9	2.4±1.6	0.64
**Number of attacks in the past year**	2.7±1.3	2.5±1.6	0.73
**Female**	25 (86.2%)	27 (93.1%)	0.38
**Positive AQP4**	15 (51.7%)	11 (37.9%)	0.38
**Positive FH**	2 (6.9%)	1 (3.4%)	0.55
**PMH**			
**Asthma**	0 (0%)	2 (6.9%)	0.17
**Evens syndrome**	0 (0%)	1 (3.4%)	
**Hypothyroidism**	6 (20.7%)	2 (6.9%)	
**Hypothyroidism, DM**	0 (0%)	1 (3.4%)	
**Migraine**	0 (0%)	2 (6.9%)	
**None**	22 (75.9%)	21 (72.4%)	
**Thalassemia**	1 (3.4%)	0 (0%)	
**DH**			
**Rituximab**	21 (72.4%)	17 (58.6%)	0.58
**Azathioprine**	7 (24.1%)	10 (34.5%)	
**Rituximab and cyclophosphamide**	1 (3.4%)	1 (3.4%)	
**Azathioprine and corticosteroid**	0 (0%)	1 (3.4%)

AQP4: Neuromyelitis optica antibody; FH: NMOSD or MS family history; PMH: past medical history; DH: drug history; DM: diabetes mellitus; EDSS: Kurtzke Expanded Disability Status Scale

Continuous variables are present as mean±SD; Qualitative variables are present as frequency (percentage)

**Fatigue:** The comparisons of fatigue score at the base, third month, as well as fatigue score changes between two study groups are presented in table 2. There was no significant difference in the fatigue severity at the baseline of study between patients in ginseng and placebo group (40.20±13.51 vs. 35.03±13.51; P-value: 0.15). By considering the base fatigue score as a confounder factor, the final fatigue score after three months of supplementation was significantly lower in the ginseng group compared with placebo group (28.97±14.18 vs. 38.79±12.27; p ˂0.01). The extent of changes in FSS before and after intervention among each group was considered as the criterion for effectiveness of supplementation and compared between two study groups. The extent of FSS change was significantly greater in the ginseng group in comparison with placebo. Note that the changes in the ginseng group were descending, while in the placebo, FSS had an ascending trend (-11.24±12.75 vs. 3.75±8.75; p ˂0.01). 

FSS scores from pre- and post-treatment were compared in each study group (table 3). The mean ± SD FSS decreased in ginseng group (from 40.21±13.51 to 28.97±14.18; p ˂0.01), in contrast to the placebo group which had an increase from 35.03±13.51 to 38.79±12.27 (P-value: 0.02). As reported in table 3, there was a significant effect by ginseng on the severity of fatigue felt by the patients.

**Safety:** Administration of oral ginseng 250 mg TDS to treat fatigue showed no adverse effects during and after the study. 

**Table 2:** Comparing the effects of supplementation on fatigue score between the two study groups

**Variables**			
**P-value**	**Placebo**	**Ginseng**	
0.15	35.03±13.51	40.20±13.51	Fatigue score base*
**˂** **0.01**	38.79±12.27	28.97±14.18	**Fatigue score after intervention** ^&^
**˂** **0.01**	3.75±8.75	-11.24±12.75	Change of fatigue score*

Data are presented as mean ± SD, *p-value is calculated using independent sample t-test, ^&^p-value is calculated using ANCOVA test by adjustment for base fatigue score

**Table 3:** Comparing the Fatigue Severity Scale before and after intervention among each study group

**FSS score**			**Variables**	
**P-value**	**After**	**Before**		
**˂** **0.01**	28.97±14.18	40.21±13.51	**Ginseng** **(Mean ± SD)**	**Intervention group**
0.02	38.79±12.27	35.03±13.51	**Placebo** **(Mean ± SD)**	

P-value is calculated by Paired sample t-test

## Discussion

This study revealed a significant effect of ginseng on reducing the severity of fatigue among NMOSD patients. The participants in the ginseng group showed a descending trend of fatigue score after three months of consumption of 250-mg ginseng TDS, but this trend was ascending in the placebo group. Fatigue is a common symptom and a major burden in neuroimmunological disorders (8). The exact mechanism of fatigue in NMOSD is poorly understood; however, metabolic diseases and disorders of neuromuscular junction transmission may play a role in this regard (7).

Management of fatigue is unclear and is considered as a challenge in NMOSD. Accordingly, a study showed that 71.4% of NMO patients feel fatigued. Moreover, Pan et al.’s study on sleep disturbances and depression established a correlation with fatigue. It has been shown that fatigue is an important predictor of quality of life (QOL), since patients with fatigue had a lower QOL. Further, the level of fatigue severity has a direct correlation with depression severity, sleep disturbance, and pain intensity (8). To the best of our knowledge, there are limited studies on the treatments of fatigue in NMOSD. There was one pilot study with seven patients, which was performed on the efficacy of tocilizumab on NMOSD, which showed some positive results regarding the reduction of EDSS and fatigue; however, this study had some differences with ours in many aspects (10). Paton in 2021 investigated the effect of satralizumab as an antagonist of interleukin-6 (IL-6) receptor for treatment of NMOSD. This paper reported the beneficial effect of Satralizumab among patients with positive anti-AQP4 antibodies. However, no beneficial effect on fatigue and pain was founded (22). Akaishi et al. in 2021, in a study on early initiation of oral prednisolone for prevention relapse, highlighted the beneficial effect of this treatment on the ensuing fatigue and depression from NMOSD (23).

Natural medicines such as ginseng could affect fatigue with their antioxidant activity (18). Studies have shown that excessive reactive oxygen species generation can lead to chronic fatigue (16, 17). Also, the main active ingredient of ginseng (ginsenoside) lessens the skeletal muscle oxidative stress (24). Based on the literature, ginseng can be considered as a safe treatment for fatigue in chronic illnesses with no major side effects. It has been revealed that ginseng can improve physical capacity while also alleviating fatigue. Further, the effects have previously been studied on MS patients (13). Accordingly, it is considered as the standard herbal treatment for fatigue (25).

Our results are in agreement with the findings of studies on multiple sclerosis. Etemadifar et al. evaluated the efficiency and safety of ginseng in MS patients. They found that ginseng is safe and effective on the treatment of fatigue in MS patients. Despite the similar results regarding fatigue severity, the EDSS diminished in their study in contrast to our study. Also, the mechanisms responsible for fatigue are poorly understood in MS, and the information is even less available on NMOSD (13). Overall, this study concluded ginseng as a possible safe and effective candidate for the treatment of fatigue in NMOSD.

It can be argued that menopause, which can affect fatigue, was not evaluated in this study, and is considered as one of the limitations of the study. Further, the interpretations of our results are limited due to short time of the follow-ups and use of simple randomization. Thus, further studies are warranted to evaluate and clarify the effects of ginseng on fatigue in NMOSD.
